# Barriers and facilitators of implementation of liver screening in an outreach-based care pathway for people experiencing homelessness and substance use disorders: a qualitative study of clinicians’ and stakeholders’ perspectives

**DOI:** 10.1186/s12954-025-01375-w

**Published:** 2026-03-02

**Authors:** Nikolaos Mylonas, Laura Hazeldine, Maria Walsh, Chris Daly, Martin Prince, Stephen J. Kaar

**Affiliations:** 1 Greater Manchester Mental Health NHS Foundation Trust, Manchester, UK; 2 Division of Nursing, Midwifery & Social Work, Faculty of Biology, Medicine and Health, University of Manchester, Manchester, UK; 3 Manchester University NHS Foundation Trust, Manchester, UK; 4 Division of Psychology and Mental Health, School of Health Sciences, Faculty of Biology, Medicine, and Health, University of Manchester, Hathersage Road, Manchester, M13 9WL UK

**Keywords:** Homelessness, Hepatology, Liver disease, Vibration-controlled transient elastography, Drugs and alcohol

## Abstract

**Background:**

People experiencing homelessness face disproportionate rates of liver disease, yet are often excluded from mainstream healthcare services. Vibration-Controlled Transient Elastography (VCTE) is a non-invasive method for liver screening increasingly used in community settings. However, evidence on implementing such screening pathways in outreach services remains limited. This study explored the views of clinicians and stakeholders on the barriers and facilitators to implementing a VCTE liver screening pathway within an assertive outreach drug and alcohol service in North West England.

**Methods:**

A qualitative design involving semi-structured interviews was used to explore the implementation of the pathway. Twenty participants, including frontline clinicians and stakeholders involved in service design, commissioning, and delivery, were interviewed. Data were analysed using Framework Analysis guided by the Consolidated Framework for Implementation Research (CFIR). Both deductive coding using CFIR domains and inductive coding were applied.

**Results:**

Key barriers included training costs, capacity within hepatology services, patients’ difficulties in accessing healthcare, and uncertainties around continuity of funding. Facilitators included the immediate feedback provided by VCTE supporting harm reduction advice, targeted initial funding for services supporting people experiencing homelessness, a strong team culture of person-centred care, staff motivation, and the flexibility of the outreach delivery model. Challenges around interpreting results and navigating referral processes highlighted the need for stronger cross-sector collaboration and workforce development.

**Conclusions:**

Integrating liver screening into outreach-based drug and alcohol services was considered acceptable by staff and stakeholders when supported by interagency partnerships, targeted funding, and motivated staff. Future implementation efforts should prioritise keyworker training and enhanced communication between drug and alcohol and hepatology services.

## Background

The UK has seen death rates due to liver disease increase by more than 400% between 1970 and 2010, in contrast with the mortality rates due to diseases in all other organs, which decreased [1]. The increase in deaths from alcohol-related liver disease in the country has been described as an ‘escalating tragedy’ [2]. People experiencing homelessness face a disproportionate burden of liver disease, with studies indicating that up to 26% have clinically significant hepatic fibrosis, primarily due to high rates of alcohol use disorder and hepatitis C virus infection, leading to increased liver-related morbidity and mortality [3, 4]. Mainstream health services tend to be ill-equipped to meet the complex needs of this population, indicating the necessity for tailored, person-centred approaches to care [5]. Vibration Controlled Transient Elastography (VCTE) is a non-invasive and user-friendly technique for the early detection of hepatic fibrosis [6]. VCTE enables a quick assessment of liver disease, offering a chance to increase patient awareness regarding potential risk factors [7]. The method quantifies liver stiffness, which is reported in kilopascals (kPa) [8]. The optimal cut-off value for diagnosing cirrhosis is around 14 kPa [9]. Fibrosis scores are typically staged from F0 to F4, where F0 indicates no fibrosis, F1–F3 indicate increasing levels of liver scarring, and F4 is consistent with cirrhosis [10]. The method demonstrates strong reproducibility and is becoming more prevalent in community settings [3]. A key factor contributing to its widespread use is that the procedure can be performed by a range of healthcare professionals—not exclusively medical doctors—provided they receive appropriate training [11]. In the UK context, the National Institute for Health and Care Excellence has recommended in two separate clinical guidelines the use of VCTE for high-risk populations, such as people with high-risk alcohol consumption and/or hepatitis C virus infection [12, 13].

VCTE has also demonstrated high acceptability across various community-based populations. In a nurse-led outreach clinic targeting patients experiencing social deprivation and alcohol use disorder, 67% of those offered VCTE accepted the test, and all individuals identified as needing further care engaged with follow-up services [8]. In Australia, a trial found that community-based VCTE screening was well received by both patients with metabolic dysfunction-associated steatotic liver disease (otherwise known as non-alcoholic fatty liver disease) and general practitioners, facilitating earlier risk stratification and reducing waiting times for specialist care [14]. Similarly, among people who inject drugs, non-invasive liver assessment was found to be both feasible and acceptable in drug and alcohol treatment settings, with high uptake reported [15]. In a study with participants on opioid substitution treatment where 40% of the sample experienced homelessness, there was a high percentage (over 90%) of attendance to the VCTE scan [16]. Similarly, in a study of people experiencing homelessness 127/131 (97%) participants accepted community-based VCTE [3]. VCTE has more recently been recognised as a potential opportunity for initiating discussions around harm reduction and facilitating referrals for individuals with substance use disorders (SUDs) within both primary care and community-based drug and alcohol services [8, 17]. These findings support the integration of VCTE into outreach and low-threshold services as an acceptable tool for engaging high-risk populations in liver health screening.

Research on the implementation of VCTE pathways in marginalised or high-risk populations remains scarce. Existing studies have largely focused on feasibility or diagnostic performance rather than on implementation processes. For example, some studies [7, 15] reported high acceptability of VCTE among people who inject drugs and in primary-care populations, but noted logistical obstacles and low awareness of liver disease among participants. Matthews et al. [8] described how a nurse-led outreach clinic increased uptake among socially deprived service users with high alcohol consumption, yet they did not report on interviews with staff and stakeholders. Similarly, the ‘VALID’ study [3] demonstrated high acceptability of VCTE among people experiencing homelessness, but did not evaluate organisational or behavioural barriers to implementation. At a system level, previous studies on non-alcoholic fatty liver disease and liver care of people with alcohol use disorder [18, 19] identified inadequate cross-speciality communication, unclear referral pathways, and the difficulty of interpreting test results as significant barriers towards wider adoption. Taken together, these studies suggest that while VCTE is acceptable and feasible in these settings, its integration into outreach or homelessness pathways is poorly understood, particularly from the perspective of professionals responsible for delivery. This study, therefore, addresses a significant gap by examining the contextual and organisational determinants of implementing VCTE in an outreach-based drug and alcohol service.

Engaging with professionals directly involved in service delivery offers critical insights into the operational realities of integrating liver screening into harm reduction settings [18–21]. Their experiences can illuminate how models of care affect patient engagement, identify structural and logistical barriers to implementation, and highlight enablers that support sustainability and scalability. This study explored clinicians’ and stakeholders’ views on the barriers and facilitators of implementing a VCTE pathway within an outreach-based drug and alcohol service targeting people experiencing homelessness and SUDs in North West England. By incorporating these perspectives, the study aims to address a key evidence gap and inform the development of integrated, community-based liver screening pathways for this cohort.

## Methods

### Design

This study employed a qualitative design to explore the views of clinicians and stakeholders on the implementation of a liver screening pathway using VCTE within an outreach-based drug and alcohol service. The study was conducted alongside the implementation of the liver screening pathway and aimed to capture clinicians’ and stakeholders’ experiences as the pathway was being embedded into routine outreach practice. The views of a sample of patients who experienced the pathway are reported in a separate publication, providing a complementary account of the screening’s acceptability and perceived impact [22].

### Setting

The liver screening pathway was developed through a partnership between local authorities and a National Health Service (NHS) mental health trust in North West England. Its purpose was to enable the early detection, communication, and clinical follow-up of liver disease among individuals experiencing homelessness and SUD—a group often underserved by primary care and frequently diagnosed with advanced liver fibrosis only during hospital admissions. The National Institute for Health and Care Excellence states in their guideline:

Using FibroScan [i.e., the device of VCTE] to assess liver fibrosis and cirrhosis outside secondary and specialist care has the potential to detect liver disease earlier. Providing tests at locations that are closer to more people who need them may improve access and attendance at appointments. This may also reduce health inequalities for people from underserved groups (such as disabled people, people living in rural areas or people from lower socioeconomic backgrounds) [13].

In this context, the screening pathway developed in this setting refers to the whole process of identifying, assessing, and linking individuals to appropriate care. It encompasses: (1) offering and conducting non-invasive liver screening using VCTE; (2) communicating and discussing the results with the patient, including tailored harm reduction advice; and (3) referring individuals with clinically significant findings (e.g., probable cirrhosis) to hepatology services for specialist assessment and ongoing management.

The pathway was embedded within a specialist assertive outreach drug and alcohol service supporting people who are experiencing homelessness or are at risk of losing their accommodation. This service provides 6–12 months of outreach-based support, including opioid substitution treatment and referrals for detoxification and rehabilitation placements. Routine dried blood spot testing for detection of hepatitis C virus is undertaken at service entry as part of the baseline clinical screening protocol. Within this model, VCTE is performed by a trained nurse as part of the routine assessment for individuals referred for SUD. The same device is also used in the nearby hospital-based drug and alcohol detoxification unit, following training delivered to medical and nursing staff. Patients with a fibrosis score consistent with cirrhosis (F4) are referred to the hepatology department of the local acute hospital, while all patients receive individualised feedback and harm reduction advice based on their screening results and overall clinical needs.

### Participants and recruitment

Participants included both clinicians of the assertive outreach service and system-level stakeholders involved in the design, commissioning, and delivery of substance misuse and hepatology services at the local and national levels. The clinicians comprised outreach keyworkers (who act as case managers providing ongoing support for housing, benefits, and engagement with treatment), nurses (who support harm reduction interventions such as opioid substitution treatment and medically-assisted withdrawal, hepatitis virus C testing, and VCTE scans), and consultant psychiatrists (who provide clinical leadership, prescribe medication, and supervise the multidisciplinary team). All these clinicians were responsible for delivering care within the specialist drug and alcohol outreach service and for implementing the liver screening pathway. Among this group, we sought to explore their experiences of delivering the intervention, perceptions of its acceptability and feasibility, and the practical challenges encountered during implementation. Stakeholders were recruited who either (1) held policy, leadership, or commissioning roles in local NHS Trusts or national bodies related to substance misuse or hepatology services, or (2) were clinicians—such as hepatologists or alcohol care nurses— who were based in acute hospital settings within the local area. In the United Kingdom, commissioners are senior public health officials or managers who plan and fund health services on behalf of local authorities or the NHS. From this group, we aimed to understand the broader contextual, organisational, and structural factors shaping the development and sustainability of the pathway, as well as their views on cross-sector collaboration. Stakeholder recruitment was facilitated through a snowball sampling approach, initiated via professional networks. Potential participants were contacted by email and provided with a participant information sheet. Interviews were scheduled at a mutually convenient time, and consent was obtained electronically. All participants were offered a £10 voucher in recognition of their time, although some declined compensation.

### Data collection

Qualitative data were collected between December 2024 and April 2025 through semi-structured interviews. All participants provided informed written consent prior to participation. Interviews were conducted by authors NM (leading) and LH via Microsoft Teams and recorded using encrypted devices supplied by the NHS organisation sponsoring the study, in accordance with information governance and data security protocols. Transcriptions were generated using the built-in ‘Record and Transcribe’ function in Microsoft Teams and were checked verbatim for accuracy.

The topic guides were developed with reference to the Consolidated Framework for Implementation Research (CFIR) [23], which offers a comprehensive approach for exploring implementation processes across multiple domains: intervention characteristics, inner setting, outer setting, characteristics of individuals, and the implementation process itself. The CFIR was chosen for its applicability to studying interventions where participants—such as healthcare professionals, service leads, and commissioners—hold influence over implementation outcomes [23]. This approach enabled the exploration of both structural and experiential dimensions of implementation, capturing a range of contextual and organisational influences on the delivery of the VCTE pathway.

### Data analysis

Interview data were analysed using the Framework Analysis approach [24], guided by constructs outlined in the CFIR. A codebook was developed in NVivo 14, based on the CFIR domains and constructs, to support deductive coding. Inductive coding was also employed to capture original themes that extended beyond the CFIR framework.

To enhance consistency and rigour, three transcripts from each participant group were coded collaboratively by two researchers to ensure consistency in the application of the framework. Coding differences were discussed until agreement was reached. All remaining transcripts were coded independently, with regular meetings to discuss discrepancies and align analytical decisions. This collaborative process ensured analytic coherence without relying on quantitative measures of coder agreement, which are not typically applied in Framework Analysis. Themes were developed through comparison of coded content, focusing on convergences and divergences in participants’ accounts of the barriers and facilitators to implementing the VCTE pathway.

### Ethics

This study was approved by the Health Research Authority (HRA) and Health and Care Research Wales (HCRW) on 7th November 2024 (REC reference: 24/SW/0089). All participants signed a written consent form to participate in this study. References to specific services and locations have been redacted.

### Reflexivity

All members of the research team had prior professional experience in the delivery of clinical care and/or research with populations affected by homelessness and SUD. Acknowledging the potential influence of these backgrounds on data interpretation, we engaged in regular team-based reflexive discussions throughout the data collection and analysis phases.

## Results

### Sample characteristics

Of the 12 staff members invited to participate, 10 accepted the invitation (eight women and two men), while two outreach keyworkers declined. Similarly, out of 12 stakeholders approached, 10 agreed to take part (four women and six men); one declined due to transitioning to a new role, and one did not respond. Sample characteristics are summarised in Table 1.

**Table 1:** Participants’ role, location and gender

Sample group	Role	Location	Gender	(*n* = 20)
Clinicians	Outreach keyworker	North West	Male	1
			Female	5
	Substance misuse nurse	North West	Male	0
			Female	2
	Psychiatrist	North West	Male	1
			Female	1
Stakeholders	Public health commissioner	North West	Male	1
			Female	0
		National	Male	1
			Female	0
	Hepatologist	North West	Male	2
			Female	2
	Inpatient detoxification unit staff	North West	Male	2
			Female	1
	Acute hospital alcohol care team nurse	North West	Male	0
			Female	1

### Barriers and facilitators of implementation

The complete list of identified barriers and facilitators of implementation is reported in Table 2.

**Table 2:** Summary of the main facilitators and barriers of implementing the screening pathway sorted under the consolidated framework for implementation research (CFIR) domains

CFIR Domain	Barriers	Facilitators
1. Innovation Characteristics	Cost of training to deliver VCTE	Immediate feedback followed by harm reduction advice
2. Outer setting	Limited capacity of hepatology services	Funding services for people experiencing homelessness
3. Inner setting	Outreach keyworkers’ knowledge regarding VCTE and liver care pathways	Team culture of person-centred care
4. Characteristics of individuals	Patients’ motivation and difficulties in accessing services	Staff recruitment and motivation
5. Implementation process	Limited planning time and rapid decision-making at early stages of implementation	Outreach model

### Innovation characteristics

Within the CFIR framework, the ‘innovation characteristics’ domain refers to the features of the intervention itself that influence its implementation, such as its perceived advantage, complexity, and adaptability [23]. In this study, participants discussed both enabling and constraining aspects of the VCTE screening pathway. The training to deliver VCTE is offered only by the company that produces the devices. Some participants noted that the cost of training staff to deliver the VCTE scan was prohibitively high (at the time of writing this manuscript, £1,350 for a three-hour session accommodating up to three professionals), limiting the number of professionals who could be trained to use the device. This constraint was particularly significant when trained staff members left their roles, thereby reducing the service’s capacity to expand liver scanning provision across additional community venues frequented by individuals experiencing homelessness and SUD. As one staff member explained:

“*But the frustrating thing was the training was extremely expensive for members of staff to do it […] It was linked to the cost because in an ideal world […] we’ve got a machine. It’d be great if all members of staff could be trained and we could […] do mobile clinics […] left*,* right and centre. But yeah*,* it was down to cost.” (Staff 7*,* nurse)*.

Conversely, an important facilitator related to the immediacy of feedback following the scan. Both groups of participants highlighted that the scan offers an opportunity to initiate conversations around harm reduction and person-centred care planning. Participants noted that the short duration of the procedure and the immediacy of the results enhance its suitability for this population. Some emphasised that the provision of a tangible score, one that can serve as either a benchmark to maintain or a target to improve, along with the imaging of the score on a screen that can be observed by the patient, increases the scan’s perceived relevance and acceptability among this cohort. As one staff member explained:

*“I think sometimes people don’t really realise the kind of damage that they’re doing to their body. When it’s shown to them on a screen and they’re given a result*,* I think it becomes a bit more real […] making people aware of the risks and the damage they’re doing to their body contributes towards people hopefully making changes to their behaviour in the future.” (Staff 5*,* outreach keyworker)*.

This observation illustrates how, according to clinicians, immediate, visual feedback can transform a biomedical test result into an emotionally salient experience that supports behaviour change.

### Outer setting

The ‘outer setting’ domain captures the external context shaping implementation, including interorganisational relationships, funding, and service capacity [23]. In this study, participants described how broader system-level factors—particularly hospital capacity and funding mechanisms—affected the feasibility of embedding liver screening into outreach services. A key barrier to implementing the VCTE pathway was the concern expressed by hepatology departments at local acute hospitals regarding a potential increase in referrals following the expansion of liver screening within the outreach drug and alcohol service. Staff reported that a crucial component of the pathway involved ensuring hepatology consultations for patients identified with advanced fibrosis (F4 score). However, due to existing pressures on the system, hepatology services initially declined to accept these referrals. Two main issues were identified: first, limited clinical capacity; and second, structural constraints within the care pathway, which required referrals to hepatology to come exclusively from primary care, rather than directly from substance misuse services.

*“They were a bit limited on staff*,* and obviously*,* they were concerned as well that if we started scanning all our clients*,* they were going to get a massive influx of patients coming through hepatology as well.” (Staff 7*,* nurse)*.

*“So after all this time*,* must have been about eight months*,* I got a meeting directly with the hepatologist and they said […] we’re actually happy to take referrals from you*,* but you need to get the GPs to do the referral because if the GPs don’t refer to the hospital trust*,* the hospital doesn’t get paid for it.” (Staff 1*,* nurse)*.

To address this, the assertive outreach team developed a workaround in collaboration with primary care. They issued formal letters to general practitioners (GPs) outlining the clinical findings, prompting the GPs to initiate appropriate referrals to hospital-based hepatology services. These accounts reveal that implementation barriers were not solely operational, but also tied to interorganisational funding rules that limited direct collaboration between services.

A key facilitator in the implementation of the VCTE pathway was the Rough Sleeping Drug and Alcohol Treatment Grant (2022–2025), a UK government initiative designed to support evidence-based drug and alcohol treatment alongside wrap-around services for individuals sleeping rough or at risk of homelessness. This grant provided four years of funding for both the assertive outreach service responsible for delivering the pathway and the procurement of the VCTE device by the local authority.

As a stakeholder explained, the grant aimed to increase treatment engagement and reduce SUD-related mortality and homelessness of this vulnerable cohort:

“*So*,* the main objectives are to support people who are rough sleeping or at risk of rough sleeping into drug and alcohol treatment*,* help reduce drug and alcohol related deaths and also help reduce homelessness through the support of services that are funded through the grant”. (Stakeholder 9*,* public health commissioner)*

### Inner setting

The inner setting domain refers to the structural and cultural features of the organisation where implementation occurs [23]. Participants highlighted how team culture, shared values, and internal knowledge influenced the delivery of the screening pathway. Outreach keyworkers reported limited confidence in assessing the clinical significance of VCTE results and expressed uncertainty about local liver care pathways. This lack of knowledge was seen as a potential barrier to engaging in effective harm reduction discussions and collaborative care planning with patients following the scan and the consultation with the nurse who administered it.

*“I don’t think many people know how the scoring works. I think we all need to Google it. Apart from the people delivering the scans*,* obviously.” (Staff 4*,* outreach keyworker)*.

Staff participants emphasised that delivering liver screening to this population was aligned with the service’s broader commitment to person-centred care, tailored to the unique needs and strengths of each patient. They described how their team provides holistic support to people experiencing homelessness through a combination of outreach visits, psychosocial interventions, opioid substitution treatment, access to detoxification and rehabilitation placements, and assistance with engagement in primary care and mental health services. Embedding liver screening within an established model of outreach-based care was described as facilitating implementation by aligning the intervention with existing workflows, staff’s experience in supporting a cohort with complex needs, and the overarching ethos of person-centred practice.

*“It’s part of a wider picture of what we’re prescribing for them*,* how often we’re having contact with them*,* what their treatment goals are*,* if they’re walking towards detoxes*,* [if they] are doing psychosocial interventions with the worker. It kind of fits into it*,* supports everything else that we’re doing with them clinically and psychosocial.” (Staff 1*,* nurse)*.

*“But the nurses are lovely. They’re engaging*,* they’re compassionate. They’re professional. They gave advice as well afterwards*,* which I think is important and harm reduction*,* you know? Yeah*,* they were fantastic.” (Staff 3*,* outreach keyworker)*.

### Characteristics of individuals

‘Characteristics of individuals’ is a sub-domain of the ‘Individuals’ domain of CFIR and focuses on the needs, capabilities, opportunities and motivations of individuals involved in implementation, either as innovation deliverers or innovation recipients [23]. Participants described how staff commitment and patient complexity both shaped the success of the intervention. Participants consistently reported that the implementation of the VCTE pathway took place within a broader context of significant structural barriers to healthcare access for this cohort. Individuals with no fixed abode often cannot receive appointment letters from hepatology services, particularly when appointments are scheduled several months after the initial screening. Limited literacy, lack of access to digital technology, and difficulties registering with or contacting primary care providers further compound challenges of engaging with services. Both staff and stakeholders noted the high level of complex needs within this population, including chronic physical health conditions, mental health difficulties, history of trauma, and pervasive experiences of stigma when engaging with mainstream services.

*“They don’t access healthcare very well*,* as we know. Partly because of stigma*,* and partly because*,* I think*,* it just doesn’t suit them. The way systems are set up just doesn’t suit them. (Staff 2*,* consultant psychiatrist)*

*“They then got to come to the hospital at a particular time. They have a chaotic life. They don’t have money for the bus fare. Where are they going to put all their sleeping equipment to keep it safe? Do they have a foot ulcer or something that is going to make it difficult for them to walk into the hospital? And you could just go on. Imagine every barrier at every single stage.” (Stakeholder 1*,* hepatologist)*.

The vivid description of this hepatologist moves beyond abstract notions of “barriers” to convey the embodied and logistical realities of homelessness, illustrating how fragmented systems amplify daily survival challenges and inhibit continuity of care.

Participants noted that while the screening can be a useful tool for initiating harm reduction conversations, its potential to drive immediate behaviour change should not be overestimated. Patients face significant challenges across multiple areas of their lives—such as insecure housing, SUD, and mental health difficulties—which shape their priorities and may limit their readiness to act on liver health concerns.

*“I really don’t think they’re likely to make big changes because you know*,* they’ll discount any benefit they get for [treating] the liver disease because it’s so far in the future.” (Stakeholder 1*,* hepatologist)*.

Staff motivation was identified as a critical facilitator in the implementation of the VCTE pathway, particularly in the face of barriers observed across other domains. The recruitment of a nurse with prior experience in delivering VCTE was instrumental in integrating the intervention into the routine operations of the assertive outreach service. This individual played a key role in establishing partnerships with the local drug and alcohol detoxification unit and acute hospital hepatology services, while also helping to overcome challenges in the onward referral process. Participants repeatedly emphasised the value of flexibility, initiative, and sustained commitment while navigating a care system of high complexity.

*“You need*,* as always with any innovation*,* motivated people who are able to deliver and sustain it.” (Stakeholder 5*,* consultant psychiatrist)*.

*“I think we’re lucky because [nurse delivering the screening] is so proactive. I had someone miss a scan when I was on leave. I was confident he was going*,* and he missed it*,* but she was like*,* ‘get him in on Friday’*,* which was only a couple of days after.” (Staff 9*,* outreach keyworker)*.

### Implementation process

The implementation process domain concerns how the intervention is put into practice, including planning, engagement, and adaptation [23]. Several participants noted that the initial implementation of the liver screening pathway was undertaken under significant time pressure. The decision to purchase and deploy the VCTE device was made quickly in response to an end-of-year funding opportunity, leaving little time for detailed planning of operational processes or referral arrangements. Stakeholders described the process as pragmatic but reactive, noting that uncertainties about clinical capacity, data flow, and care pathways had to be resolved after implementation began. As one commissioner explained:

*“We were trying to balance the urgency of spending the money while we had the opportunity at the end of the financial year with very little time to actually find a device and make the purchase […] We didn’t want to buy something that was never going to be used.” (Stakeholder 8*,* commissioner)*.

The absence of an initial implementation plan meant that key logistical and governance issues, such as patient follow-up and communication with hepatology services, were addressed retrospectively as challenges emerged. This demonstrates how reactive, opportunity-driven implementation, which is common in real-world innovation contexts, can produce downstream uncertainty in governance and data flow. It reflects the tension between short-term funding cycles and the long-term coordination required for sustainable service change. Currently, for example, the arrangement of the delivery of this pathway in one of the two localities of our study is uncertain, as a new tender process for addiction services has started.

A key innovation of the pathway was the delivery of VCTE screening on an outreach basis. Screenings were conducted in locations tailored to the needs of the patient, including: (a) their temporary accommodation if they were housed in a hostel for people experiencing homelessness, (b) their preferred community venues, (c) during their drug and alcohol detoxification admission and (d) in some cases, mutually agreed locations with transport support from their outreach keyworker following an updated risk assessment. This approach was seen to reduce logistical barriers and improve convenience for patients who often struggle to visit the premises of healthcare services due to social anxiety, stigma, and fear of meeting peers.

Staff emphasised that coordinating the scan with other pre-planned appointments—such as opioid substitution prescribing, detoxification and rehabilitation assessments, and physical health reviews—was a key strategy for increasing attendance and engagement.

*“What better opportunity to bring that scan to that patient rather than that patient in the community going for a scan? It’s unlikely to happen [the patient to attend the appointment without outreach support] most of the time anyway.” (Stakeholder 2*,* alcohol nurse)*.

*“So yesterday we went to do a prescribing review of a client. We’ve done a scan. We’ve got loads done just by going out into the hostel. So*,* it is a lot better going out with it [the device].” (Staff 10*,* outreach keyworker)*.

## Discussion

Harm reduction approaches for people experiencing homelessness are necessary, acceptable and beneficial [25–27]. VCTE has recently been considered as an opportunity to initiate conversations about harm reduction and onward referral for people with SUDs in primary care and in community drug and alcohol services [8, 17, 27–30]. However, little was known about the barriers and facilitators of implementing a VCTE pathway in an assertive outreach drug and alcohol service for people experiencing homelessness. This qualitative study sought to address this gap through semi-structured interviews with clinicians and stakeholders involved in service delivery and pathway development in North West England.

Findings highlight the importance of strong partnerships and connections between local government, community drug and alcohol services and acute hospitals in creating new care pathways for patients who are often excluded from mainstream healthcare systems. Such partnerships reflect the outer setting constructs of the CFIR framework, emphasising how interorganisational alignment and communication can determine implementation success. A similar study has shown that hepatology and addiction services frequently operate in silos [19] and our data illustrate how motivated individuals can bridge these structural divides. This is particularly salient given recent evidence from a national cross-sectional follow-up survey indicating that up to 36% of integrated care systems in the UK currently lack dedicated liver care pathways, with substantial regional variation among those that do [21]. A context of fragmented and siloed care systems undergoing continuous transformation can likely explain why clinicians in the assertive outreach service described a lack of confidence in describing care pathways to their patients. Regular cross-disciplinary training and structured knowledge-sharing between drug and alcohol services and hepatology teams are essential for bridging the persistent disconnect between these domains [31, 32]. Hepatologists often have limited awareness of addiction treatment pathways, while clinicians in drug and alcohol services may lack familiarity with liver disease management and referral processes [19]. Strengthening mutual understanding across these clinical disciplines can enhance collaborative care, reduce the risk of referral bottlenecks and support more informed decision-making among patients whose health literacy can be limited [33, 34]. In the pathway described in this study, these outer setting barriers to implementation were addressed by facilitators identified in other domains, such as the recruitment of individuals with the knowledge and motivation to persist in developing the pathway (characteristics of individuals domain) and a person-centred organisational culture (inner setting domain).

Although all participants considered VCTE as an acceptable opportunity to have conversations about harm reduction with people experiencing homelessness, they indicated that, given the high complexity of needs experienced by this cohort, the screening should be perceived as an aid which, combined with psychosocial interventions such as motivational interviewing, can enhance the motivation of patients to reduce risk behaviours rather than an intervention with drastic effects. In a recently concluded feasibility randomised controlled study, an association was found between knowledge of hepatic fibrosis and reduced alcohol consumption and engagement with treatment services [35]. The suggestion that the visual imaging of the score on the screen of the device can have a significant impact on patients’ motivation repeats an opinion expressed in another study of clinicians working with VCTE in a primary care setting [21] and indicates that visual-based feedback can be a target for developing psychosocial interventions for SUD in line with similar methodologies used in efforts to promote smoking cessation, physical activity and sun protection [36, 37]. Both staff and stakeholders also agreed that the fibrosis score provides a tangible biomarker that can be easily and quickly communicated to patients, serving as a focal point in their recovery journey. This aligns with findings from a previous study on the use of VCTE to assist harm reduction in community drug and alcohol services [17].

The offer of screening as part of an assertive outreach service was deemed instrumental in enhancing the acceptability of the scan among patients. The well-documented barriers that people experiencing homelessness face in accessing healthcare services [38] combined with a sense of being ‘misunderstood’ by healthcare providers [20] indicates that integrating a liver care pathway into an outreach-based drug and alcohol service is considered a promising and legitimate approach to initiate harm reduction conversations and assist in early detection of hepatic fibrosis in a cohort that otherwise would present to acute hospitals with advanced-stage liver disease. This also reinforces the significance of considering the ‘inner setting’ and ‘implementation process’ domains in CFIR, where flexible service delivery, relational continuity, and staff commitment can offset systemic deficiencies. The outreach model effectively repositions liver screening from a specialist hospital-based activity to a person-centred encounter embedded in existing relationships of trust. Outreach interventions have previously been found to promote increased access to health care services for people experiencing homelessness [39]. A key implication emerging from our findings is that combining VCTE screening with other appointments that are possibly perceived by the patients as more urgent, such as opioid substitution prescribing or assessment for detoxification and rehabilitation, may be an effective strategy to improve engagement with harm reduction initiatives and deliver more holistic, person-centred care during outreach visits mutually agreed by the clinician and the patient. While the outreach keyworkers were highly motivated and played a crucial role in engaging patients with the screening process, which was emphasised throughout the interviews, some expressed limited confidence in interpreting VCTE scores and navigating liver care pathways. Staff delivering the scans had access to written interpretation guides and were trained to provide clear feedback and indicate when onward referral was required. They operated with senior clinical support. In cases of uncertainty, they could seek clarification from senior clinicians to ensure accurate communication of results and follow-up planning. The issue described by keyworkers likely reflects a broader training need among staff who were not directly involved in performing the scans, but who later encountered the results in patient notes and discussed the results during case management sessions. This is characteristic of an early implementation phase, when knowledge of new pathways is still being embedded across multidisciplinary teams. A separate but related challenge concerned the outreach staff’s awareness of onward referral routes. The VCTE score clearly indicates the clinical next steps; however, the pathways into hepatology services are themselves fragmented and, at times, changing. This structural factor, rather than uncertainty about interpreting VCTE results, contributed most to the reported difficulty of outreach keyworkers in explaining follow-up pathways to patients. Moreover, in the UK, there are no nationally agreed professional standards for drug and alcohol keyworkers, meaning that individuals in these roles often come from diverse educational and experiential backgrounds. This variability may contribute to differing levels of confidence in explaining results or advising on follow-up care, particularly during the early stages of implementation when training and supervision structures are still being embedded.

Targeted interprofessional training and shared case discussions involving GPs, hepatology, and addiction services could help strengthen the bridge between different clinical specialties. Previously reported attempts towards this include an alcohol-related liver disease multi-stakeholder hub and joint clinics organised by consultant hepatologists and addiction nurses in a hospital setting [33, 40]. As highlighted by Winder et al. [31], developing “expanded alcohol-associated liver disease care” models requires equipoise between biomedical and psychosocial approaches, sustained interprofessional collaboration, and ongoing relational continuity with patients and families. Integrating these principles into outreach-based drug and alcohol services could enhance coordination and ensure that liver screening initiatives evolve beyond stand-alone interventions toward longitudinal, collaborative care frameworks.

Finally, the study’s findings highlight broader questions of sustainability. While the Rough Sleeping Drug and Alcohol Treatment Grant provided crucial financial and organisational support, participants expressed uncertainty about continuation and care delivery beyond the funding period. Sustained commissioning arrangements and policy-level commitment are needed to embed such pathways into routine care, supported by mechanisms for ongoing workforce development, data sharing, and evaluation.

### Strengths and limitations

This study has several strengths. It is among the first to examine the implementation of a VCTE liver screening pathway within an assertive outreach drug and alcohol service specifically targeting people experiencing homelessness. By drawing on the CFIR and incorporating the perspectives of both frontline staff and stakeholders, the study provides a comprehensive understanding of the contextual and organisational factors that shape implementation. The use of an implementation science lens enhances the relevance of the findings for informing future pathway development. However, the study also has limitations. It was conducted in a single geographic area and service configuration, which may affect the transferability of findings to other settings with different commissioning arrangements or service infrastructures. While this article focuses on staff and stakeholder perspectives, the views and experiences of patients are reported in a separate publication arising from the same research project, providing a complementary account of pathway acceptability and perceived impact [22].

## Conclusions

This study highlights the value of delivering liver fibrosis screening through outreach, which can enhance engagement with harm reduction among people experiencing homelessness and SUDs. Integrating VCTE into existing outreach drug and alcohol services was viewed as acceptable and feasible by clinicians and stakeholders. Strong partnerships and connections between local government, community services, and acute hospitals were identified as essential for implementation, facilitated by clinicians who were motivated to deliver person-centred care to a cohort with largely unmet needs. The findings support the expansion of such services, and further research is needed to investigate the long-term effects of these pathways.

## Data Availability

No datasets were generated or analysed during the current study.

## Abbreviations

ARC-GM: Applied Research Collaboration Greater Manchester
CFIR: Consolidated Framework for Implementation Research
GP: General Practitioner
HRA: Health Research Authority
HCRW: Health and Care Research Wales
kPa: kilopascals
MAHSC: Manchester Academic Health Science Centre
NHS: National Health Service
NIHR: National Institute for Health and Care Research
SUD: Substance Use Disorders
UK: United Kingdom
VCTE: Vibration Controlled Transient Elastography
